# Prognostic impact of *MYH9* expression on patients with acute myeloid leukemia

**DOI:** 10.18632/oncotarget.10613

**Published:** 2016-07-15

**Authors:** Mengxia Yu, Jinghan Wang, Zhijuan Zhu, Chao Hu, Qiuling Ma, Xia Li, Xiufeng Yin, Jiansong Huang, Ting Zhang, Zhixin Ma, Yile Zhou, Chenying Li, Feifei Chen, Jian Chen, Yungui Wang, Hanzhang Pan, Dongmei Wang, Jie Jin

**Affiliations:** ^1^ Department of Hematology, The First Affiliated Hospital, Zhejiang University College of Medicine, Hangzhou, China; ^2^ Institute of Hematology, Zhejiang University, Hangzhou, China; ^3^ Key Laboratory of Hematologic Malignancies, Diagnosis and Treatment, Zhejiang, Hangzhou, China; ^4^ Department of Hematology, Fujian Medical University Union Hospital, Fuzhou, China; ^5^ Department of Hematology, The Second Affiliated Hospital of Henan University of Traditional Chinese Medicine, Zhengzhou, China

**Keywords:** MYH9, acute myeloid leukemia, prognosis

## Abstract

*MYH9* expression has previously been demonstrated as an independent predictor of clinical outcome in solid tumors. However, the prognostic relevance of *MYH9* expression in acute myeloid leukemia is still unclear. Here, we found high *MYH9* expressers were seen more frequently in females and more frequently in M4 morphology. We also found high *MYH9* expressers had lower percentage of bone marrow blasts. In addition, overexpression of *MYH9* was associated with an inferior overall survival. Notably, distinct microRNA signatures were seen in high *MYH9* expressers. These results were also validated in an independent cohort of AML patients using the published data. In conclusion, gene of *MYH9* expression might serve as a reliable predictor for overall survival in AML patients.

## INTRODUCTION

Acute myeloid leukemia (AML) is a heterogeneous group of hematologic malignancies characterized by a wide spectrum of prognostically relevant cytogenetic aberrations, oncogenes of mutations and/or abnormal expressions. To date, although chromosomal abnormalities together with *NPM1*, *FLT3-*ITD, and *CEBPA* mutations have been recommended as an effective tool for the risk stratification [1], the reliable prognostic biomarkers for personalized therapy are still required.

In our phase 3 clinical trial, we found favorable cytogenetics can serve as a survival predictor for patients with AML when treated with homoharringtonine (HHT)-based chemotherapy [2]. However, the reason why patients would benefit from HHT-based chemotherapy remains unclear [3]. We and other groups have tried to further investigate the underlying mechanisms. Recent studies suggested HHT could regulate several oncogenes expression and induce blast cells death or apoptosis [3, 4]. As far as we known, HHT has multiple drug targets. Ying Gu et al. reported that p-eIF4E was a drug target of HHT [4]. Instead, we found myosin-9 was one of the critical targets in AML cell lines. Our study further suggested HHT could up-regulate the myosin-9 expression in AML cell lines, and up-regulated myosin-9 expression was associated with the percentage of apoptotic cells treated by HHT. Thus, up-regulated myosin-9 may increase the sensitivity of the leukemia cells to the cytotoxicity of HHT *in vitro*. Taken together, these results indicated over-expressed *MYH9* might associate with favorable prognosis in leukemia. However, these results are from experiments *in vitro*, and may not always be in line with the results *in vivo*. Thus, it is urgent for us to explore the prognostic value of *MYH9* expression in AML.

The *MYH9* gene encodes the non-muscle myosin heavy chain IIA (NMMHC-IIA), a cytoskeletal contractile protein. Several mutations in the *MYH9* gene lead to premature release of platelets from the bone marrow, macrothrombocytopenia, and cytoplasmic inclusion bodies within leukocytes. It is worthy to note that myosin-9 plays a key role in cancer cell migration, invasion, and metastasis in solid tumors [5]. With respect to expression value of *MYH9*, it was reported that reduced expression of myosin-9 was exhibited in CLL samples from high-risk patients. In contrast, high *MYH9* expression was associated with poor clinical outcome in patients with several tumors such as lung cancer [6], esophageal [7], bladder [8], gastric cancer [9] and malignant pleural mesothelioma [5]. However, the clinical significance of *MYH9* expression in AML is still unclear. Here, we found AML patients with high *MYH9* expression had a distinct microRNA signature and poor overall survival. The prognostic value of *MYH9* expression was also validated in an independent cohort of AML patients.

## RESULTS

### Characteristics of patients with *MYH9* overexpression

The median expression value of *MYH9* in our cohort was 0.64 with the range from 0.02 to 4.87. Of 324 patients, 131 (40%) were classified as high *MYH9* expressers using Cutoff Finder software analysis. Clinical characteristics of patients with high *MYH9* expression are described in Table 1. High *MYH9* expressers were seen more frequently in females (55% vs. 40%, *P* = 0.008), and more frequently in M4 morphology (*P* = 0.005). We also found high *MYH9* expressers showed lower percentage of bone marrow blasts (median: 58% vs. 72%, *P* < 0.001). There was no statistically significant correlation between *MYH9* expression and other variables including age, white blood cell counts (WBC), hemoglobin levels, platelet counts, karyotype risk groups, genes mutations in *FLT3-*ITD, *NPM1* and *CEBPA*, and different treatment protocols (Table 1).

**Table 1:** Characteristics of AML patients by high and low *MYH9* expression

Variables	Low expression	High expression	*P* value
Number, (%)	193 (60)	131 (40)	
Age, median (range), years	44 (14,84)	46 (16,79)	0.262
Female, *n* (%)	77 (40)	72 (55)	0.008
WBC, median (range), × 10^9/L^1^	26 (0.3,358)	23 (0.2,230)	0.442
HB, median (range), g/L^2^	82 (38,137)	79 (42,141)	0.11
PLT, median(range), × 10^9/L^3^	41 (3,778)	40 (2,556)	0.913
BM blast,median (range), %^4^	72 (20,98)	58 (21,96)	< 0.001
FAB classification,*n* (%)^5^			0.005
M0	10 (5)	9 (7)	
M1	23 (12)	10 (8)	
M2	96 (50)	50 (38)	
M4	6 (3)	18 (14)	
M5	53 (28)	41 (31)	
M6	5 (3)	3 (2)	
Karyotype risk, *n* (%)			0.549
Favorable	7 (4)	3 (2)	
Intermediate	175 (91)	117 (89)	
Unfavorable	11 (6)	11 (8)	
Genes mutations, *n*(%)			
*FLT3ITD*	31 (17)	25 (20)	0.447
*NPM1*	43 (23)	22 (17)	0.199
*CEBPA*^*DM*^^6^	26 (15)	12 (10)	0.201
Treatment protocols^7^			0.526
DA	52 (27)	29 (22)	
IA	66 (34)	44 (34)	
HAA	75 (39)	58 (44)	

Abbreviations:

1 WBC, white blood cell;

2 HB, hemoglobin;

3 PLT, platelet counts;

4 BM, bone marrow;

5 FAB, French–American–British classification systems;

6 DM: Double-allele.

7 The protocols used for induction therapy in different groups including donorubicin/Ara-C (DA)-based treatment group, idarubicin/Ara-C (IA)-based, and homoharringtonine/Ara-C/aclarubicin (HAA)-based treatment group.

### Association of *MYH9* expression with overall survival from the ZIH cohort

**Figure 1:**
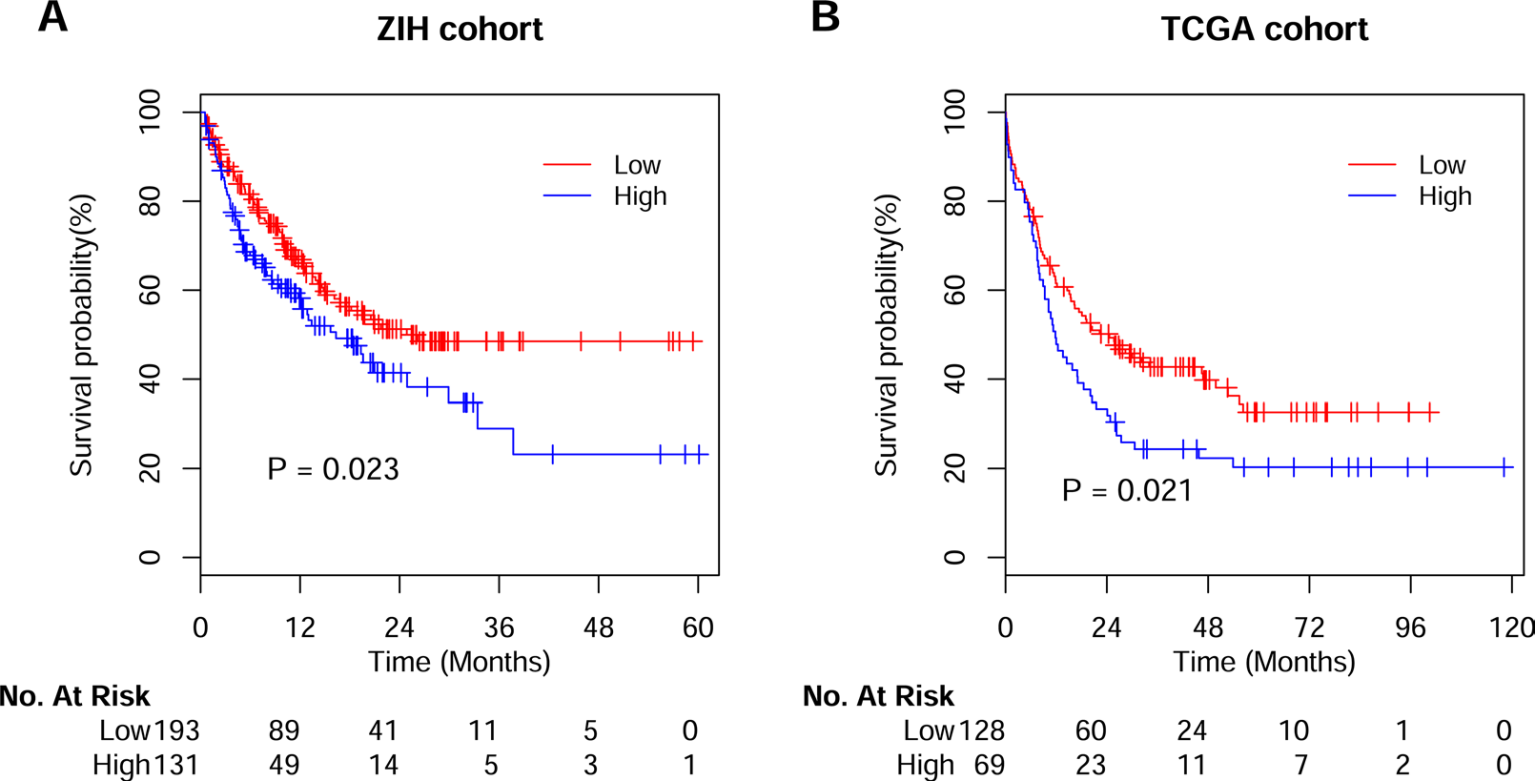
Kaplan-Meier survival analysis of AML patients Overall survival curves of the patients in ZIH cohort (**A**) and TCGA cohort (**B**) according to distinct expression value of *MYH9* gene.

With a median follow-up for living patients of 434 days (95% CI, 479–594 days), high *MYH9* expressers (*n* = 131) had an adverse OS comparing to low expressers (*n* = 193) (Figure 1A). Importantly, in the subgroup analyses we found high *MYH9* expressions were associated with poor OS in patients with the cytogenetic intermediate risk group (*P* = 0.042) and cytogenetically normal AMLs (*P* = 0.031; Figure S1A–S1B). In the multivariable analysis for OS, high *MYH9* expression was still associated with poor survival after adjusting for age, sex, WBC, percent blasts, karyotype risk groups, genes of *FLT3-*ITD, *NPM1* and *CEBPA* mutations, and treatment protocols [HR (95% CI), 1.50 (1.03, 2.19); *P* = 0.034; Table 2].

**Table 2:** Multivariable analysis for overall survival in AML patients from ZIH cohort

Variables	HR (95% CI)	*P* value
*MYH9* expression (High vs.Low)	1.50 (1.03,2.19)	0.034
Age	1.01 (1.00,1.02)	0.054
Sex (Female vs.Male)	0.81 (0.56,1.16)	0.248
WBC^1^	1.003 (1,1.006)	0.025
Percent blast	1.00 (0.99,1.01)	0.529
Karyotype-risk group		
Intermediate vs. favorable	4.63 (0.64,33.53)	0.129
Poor vs. favorable	13.31 (1.69,105.01)	0.014
Gene mutations		
*FLT3ITD*	1.45 (0.92,2.28)	0.108
*NPM1*	0.86 (0.54,1.37)	0.534
*CEBPA^DM^^2^*	0.31 (0.15,0.64)	0.002
Treatment protocols^3^		
HAA vs.DA	0.90 (0.56,1.44)	0.656
IA vs.DA	0.83 (0.54,1.30)	0.418

Abbreviations:

1 WBC,white blood cell;

2 DM: Double-allele.

3 The protocols used for induction therapy in different groups including homoharringtonine/Ara-C/aclarubicin (HAA)-based treatment group, donorubicin/Ara-C (DA)-based treatment group, and idarubicin/Ara-C (IA)-based; CI, confidence intervals; HR, hazard ratio.

### Validation of the impact of *MYH9* expression on OS from the TCGA cohort

In order to validate the prognostic value of *MYH9* expression derived from our discovery cohort, similarly, we defined high and low *MYH9* expressers from TCGA cohort using Cutoff Finder. As a result, out of 197 patients from the TCGA cohort, 69 (35%) were defined as high *MYH9* expressers and 128 (65%) as low expressers (Figure 1B). In the univariate analysis, patients with high *MYH9* expressers obtained a higher risk of death compared with lower expressers [HR (95% CI), 1.50 (1.06, 2.13); *P* = 0.02]. Moreover, in the multivariate analysis, high *MYH9* expressers were significantly associated with poor OS [HR (95% CI), 1.69 (1.17, 2.43); *P* = 0.005, Table S1] in the context of age, sex, WBC, percent blasts, karyotypes and genes of *FLT3-*ITD, *NPM1* and *CEBPA* mutations.

### MicroRNA expression profiling

**Figure 2:**
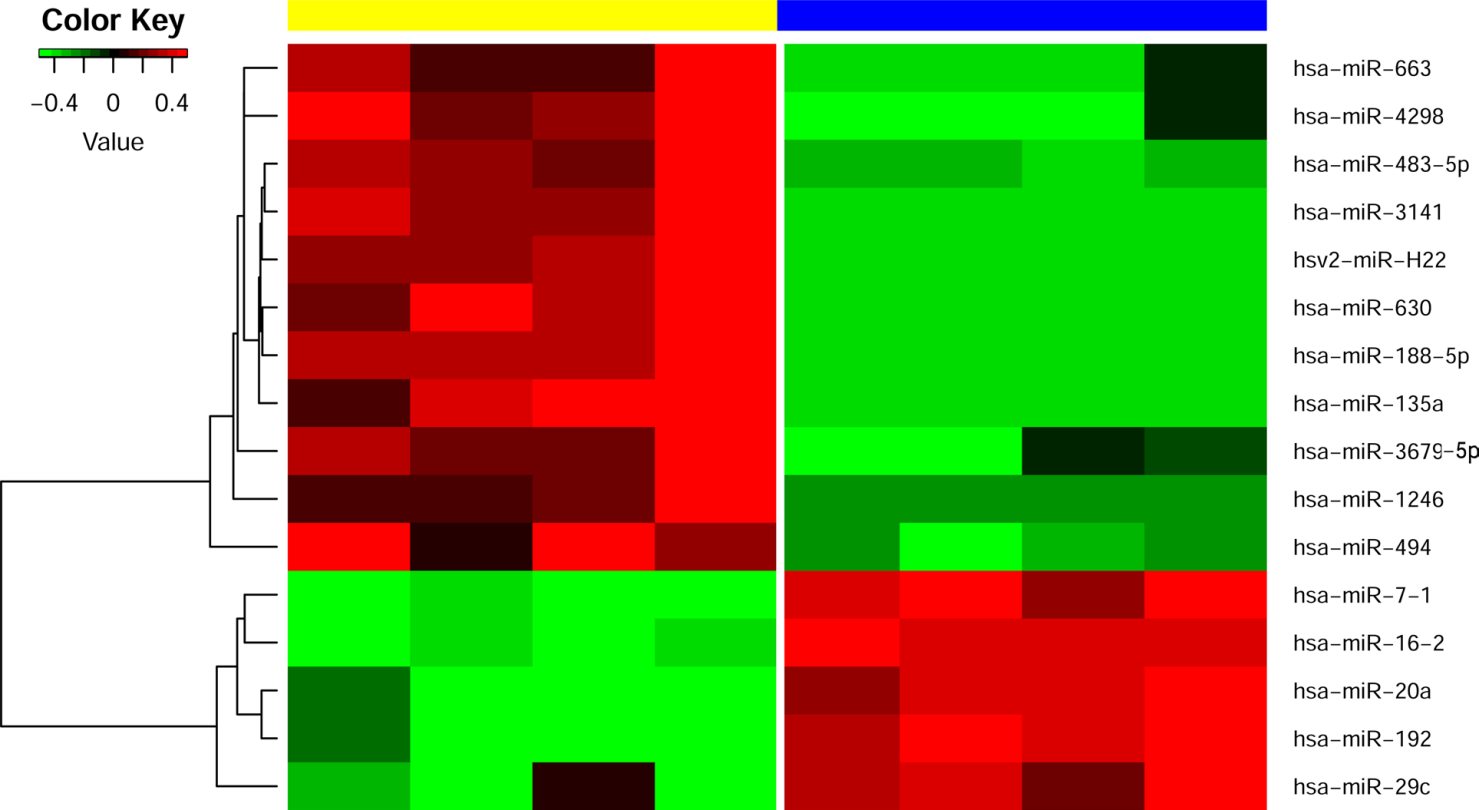
Heatmap visualizing the differentially expressed microRNAs between high and low *MYH9* expressers *MYH9* expressers are color-coded: a yellow bar indicates overexpression and blue bar under-expression in our patients with AML.

**Figure 3:**
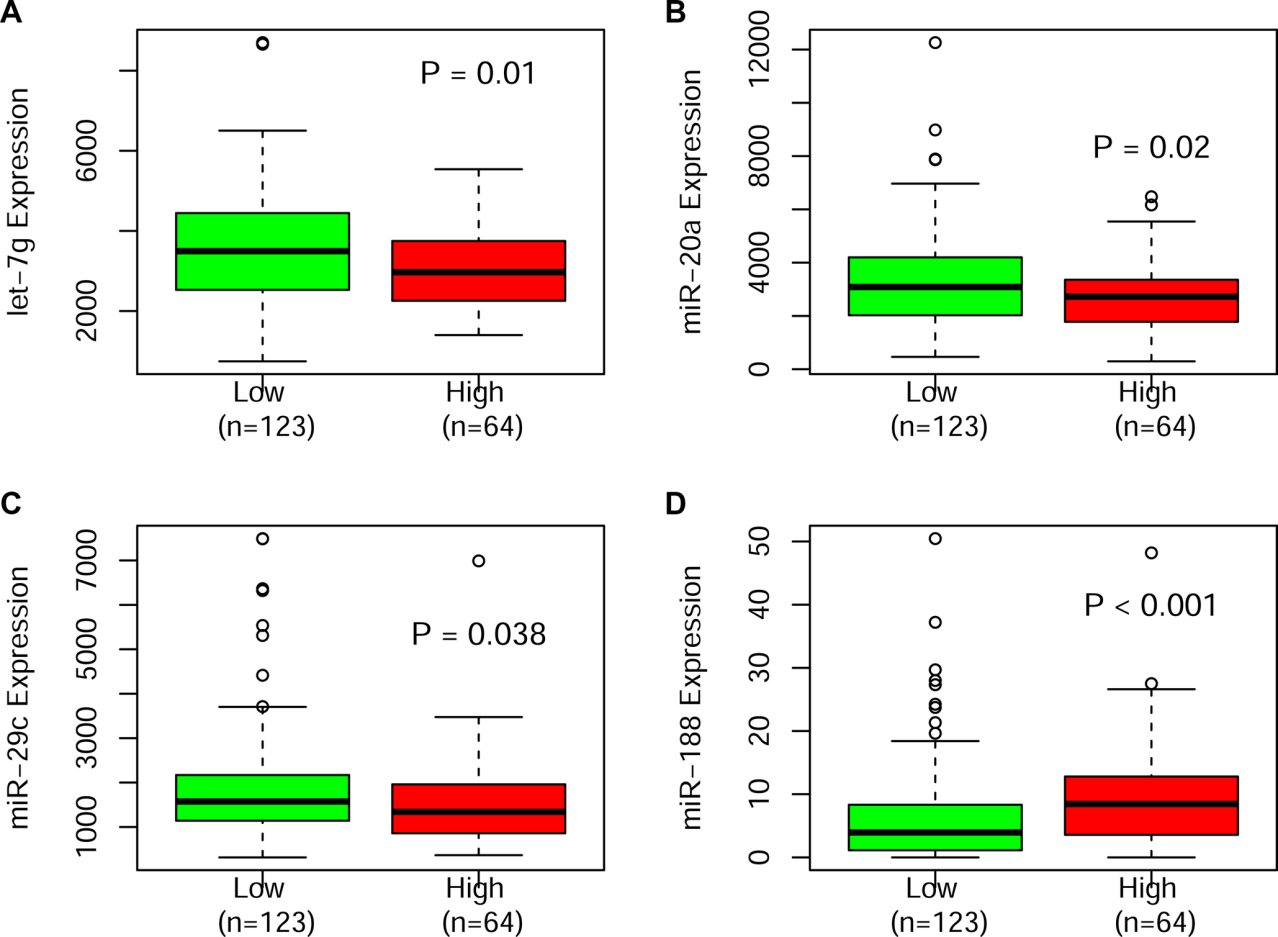
Boxplots illustrating the significantly different expression of miRNAs validated in the TCGA cohort

We used four samples with high *MYH9* expression and four samples with low expression to assess the differences of microRNA expression. The most significant changes of miRNAs in high expressers included up-regulation of miR-663, miR-4298, miR-483-5p, miR-3141, miR-630, miR-188-5p, miR-135a, miR-3679-5p, miR-1246, miR-494 and hsv2-miR-H22 and down-regulation of let-7-1, miR-16-2, miR-20a,miR-192 and miR-29c (*p*-value less than 0.005, Figure 2). Notably, we also validated up-regulated miR-188 and down-regulated miRNAs of let-7, miR-20a and miR-29c that were significantly associated with high *MYH9* expressers in a large and independent cohort of TCGA patients (Figure 3).

## DISCUSSION

In this study, we reported that high *MYH9* expression associated with shorter overall survival in AML. In addition, patients with high *MYH9* expression had distinct microRNAs. Our findings were also validated in an independent cohort of AML patients. These results suggest that *MYH9* expression will be a reliable biomarker in the clinical practice.

*MYH9* gene is located on chromosome 22q13.1 and encodes a conventional non-muscle myosin (myosin-9). Myosin-9 is one part of the myosinIIA protein. There are three forms of myosin II, called myosin IIA, myosin IIB and myosin IIC. The normal function of the myosin II protein is cytokinesis, cell motility and maintenance of cell shape [10]. The clinical significance of *MYH9* gene lesions currently are undergoing study. For example, *MYH9* gene polymorphisms are associated with cerebrovascular disease or nephropathy in patients with diabetes [11, 12]. The gene mutation in *MYH9* is involved in the development of the disorders of inherited macrothrombocytopenias. It was also reported *MYH9* expression is associated with inflammation of gastrointestinal diseases [13]. Notably, myosin-9 has been reported to be a novel tumor suppressor and play important roles in cancer progression. In this study, we found high *MYH9* expressers were linked to M4 morphology. Blasts with FAB-M4 morphology often present extramedullary disease such as cutaneous involvement. Interestingly, high *MYH9* expressers were mostly seen in patients with low percent blasts. These findings imply that high *MYH9* expression might enable blasts to migrate away from bone marrow and subsequently reside in other tissues, and escape from chemotherapy. Therefore, AML cases with high *MYH9* expression might be more resistant to chemotherapy, and associate with a poorer outcome. Here, we find that high *MYH9* expressers harbored poor overall survival in two different cohorts. These results were consistent with several recent reports showing that high expression of *MYH9* was correlated with short survival in solid tumors [7, 9, 10].

In order to further identify the biological insights into the aberrant *MYH9* expression, we conducted microRNA analysis in AMLs. Among differentially expressed miRNAs, we found 16 miRNAs were dysregulated expression in our patients. Up-regulated expression of miR-188 and down-regulated expression of 3 miRNAs including miR-20a, miR-29c and let-7 were validated in a large cohort of patients. Among these significant microRNAs, most of them have been proven to have an impact on clinical prognosis of AML patients as previous reported. For example, low miR-188-5p expression was associated with longer overall survival and event free survival for CN-AML [14]. miR-16-1 expression was used as a good candidate for prognosis prediction in chronic myeloid leukemia [15]. In addition, miR-29c is of important prognostic value and influences response to azacitidine treatment in older AML patients [16]. These differentially expressed microRNAs may help us to further understand the biological insights of poor survival of patients with high *MYH9* expression, and also serve as potential therapy targets in these patients in the future.

In conclusion, we present high *MYH9* expression as a reliable and powerful prognostic factor for patients with AML.

## MATERIALS AND METHODS

### Patients

Clinical data were abstracted from medical records of AML patients in Zhejiang Institute of Hematology (ZIH) in China. Between March 2010 and June 2014, 324 patients with detailed diagnoses and treatment information were enrolled in this study. WHO classification, conventional cytogenetic banding assay, and molecular analyses were performed as previously described in AML diagnosis [17]. Cytogenetic groups of patients were classified as favorable, intermediate, and unfavorable risk according to the NCCN guideline [18]. Favorable subgroups included t(8;21)/*AML1-ETO* and inv16/*CBFβ-MYH11*; adverse consisted of t(9;22), inv(3)/t(3;3), -5, -7, del(5q), del(7p), 11q23 and complex translocations; intermediate subtype contained cytogenetically normal and AML with other cytogenetic abnormalities. Patients received HAA (homoharringtonin 2 mg/m^2^/day for 7 days, cytarabine 100 mg/m^2^/day for7 days and aclarubicin 20 mg/m^2^/day for 5 days), DA (daunorubicin 45 mg/m^2^/day for 3 days and cytarabine 100 mg/m^2^/day for 7 days) and IA regimen (idarubicin 8–10 mg/m^2^/day for 3 days and cytarabine 150 mg/m^2^/day for 7 days) [2, 19]. In the consolidation therapy, younger patients were treated with a high-dose cytarabine-based chemotherapy [19]. The chemotherapy consolidation for elderly patients was decided by the physicians in an individualized manner, as described previously [19]. No patient in our study received allogeneic transplantation. Patients with secondary AML or acute promyelocytic leukemia were excluded. All of the subjects were well informed about the study and provided written informed consent to participate in the study. We used a AML cohort of 197 patients from TCGA (https://tcga-data.nci.nih.gov/tcga/) as a validation cohort, which contains publicly available data of gene microarray expression and clinical information [20]. The study was approved by the Institutional Review boards of the First Affiliated Hospital of Zhejiang University.

### Cytogenetic and gene mutation analysis

The bone marrow (BM) samples of de novo AML patients were studied mostly by R-banding analysis. Chromosomal abnormalities were described according to the International System for Human Cytogenetic Nomenclature [21]. DNA and RNA samples of AML patients were obtained from mononuclear cells isolated by Ficoll gradient centrifugation from bone marrow samples at primary diagnosis. Gene mutations of *NPM1*, *FLT3-*ITD, and *CEBPA* were analyzed by whole-gene sequencing as previously described [22]. RNA samples were used to determine *PMLRARA, AML1ETO*, and *CBFβMYH11* fusion genes by reverse transcription polymerase chain reaction (RT-PCR).

### Quantitative reverse transcriptase-PCR

RNA was extracted using RNeasy Mini kit (Qiagen, Venlo, Netherlands) and first-strand complementary DNA synthesis was performed using the MMLV systems (Life Technologies). Quantitative PCR was performed in triplicate using SYBR-Green PCR Master Mix kit (Takara, Japan) on an IQ5 real time PCR instrument (Bio-Rad, USA), using standard settings: 95°C (1 min), 40 cycles of 95°C (5s) and 60°C (1 min). mRNA levels were normalized to *GAPDH* housekeeping gene. The following primers were used for quantitative PCR: *MYH9* 5′-TTCAGCTCGGCAACATCGTCT-3′ (sense) and 5′-ATTCCTCTGGTGAAATCGGTCA-3′ (antisense); *GAPDH* (control), 5′-ATGGGGAAGGTGAAGGTCG-3′ (sense) and 5′-GGGTCATTGATGGCAACAATATC-3′ (antisense). PCR reactions were performed in a total volume of 25 μl containing of 1 μl of 100 ng/μl sample cDNA, 12.5 μl of 2 × PCR Mix, 1 μl of 0.5 μM of each primer, and 10.5 μl of ddH2O.

### MicroRNA experiments

For miRNA profiling, total RNA was extracted and purified using mirVana™ miRNA Isolation Kit (Ambion, Austin, TX, US) following the manufacturer's instructions. RNA intergrity number (RIN) was assessed by an Agilent Bioanalyzer 2100 (Agilent technologies, Santa Clara, CA, US). miRNA expression was performed using the Agilent Human miRNA Microarray Kit Version 16.0. Total RNA (100 ng) was hybridized per sample and processed according to the manufacturer's instructions. The arrays were scanned by an Agilent Technology G2565BA scanner. The scanned images were gridded and analyzed with Agilent Feature Extraction Software Version 10.7. Raw data were normalized by quantile algorithm, Gene Spring Software 11.0. Each microRNA signature is represented by the average of its expression value of replicate probes. Nonparameter *T*-test was used to test for the difference of microRNA signatures between high and low *MYH9* expressers. *P*-values < 0.005 demonstrated statistical difference. Hierarchical clustering based on expression levels of these microRNAs was performed and visualized by heatmap.

### Definition of clinical end points and statistical analysis

Patient characteristics were summarized using descriptive statistics, which included frequency counts, median, and range. The primary end point of the study was overall survival (OS). OS was measured as time from disease diagnosis to death from any cause, or censoring for patients alive at their last known date of contact. Determination of optimal cutoff value for *MYH9* expression in our discovery and the validation TCGA cohort was done with Cutoff Finder using log-rank test (http://molpath.charite.de/cutoff/). The proportional-hazards assumption was checked for each variable before fitting Cox models. Variables with a *p*-value less than 0.2 were selected as adjustment covariates into the multivariable analyses. All statistical analyses were conducted with R statistic packages, version 2.15.0 (www.r-project.org). The two-sided level of significance was set at *p*-value less than 0.05.

## SUPPLEMENTARY MATERIALS


