# Supplementary material for: Prognostic impact of MYH9 expression on patients with acute myeloid leukemia
Source: Oncotarget. 2016 Jul 15;8(1):156–63. doi: 10.18632/oncotarget.10613 (PMC5352077; doi:10.18632/oncotarget.10613)
Supplement: Supplementary file 1 [file oncotarget-08-156-s001.pdf]

Prognostic impact of *MYH9* expression on patients with acute myeloid leukemia

Supplementary Materials

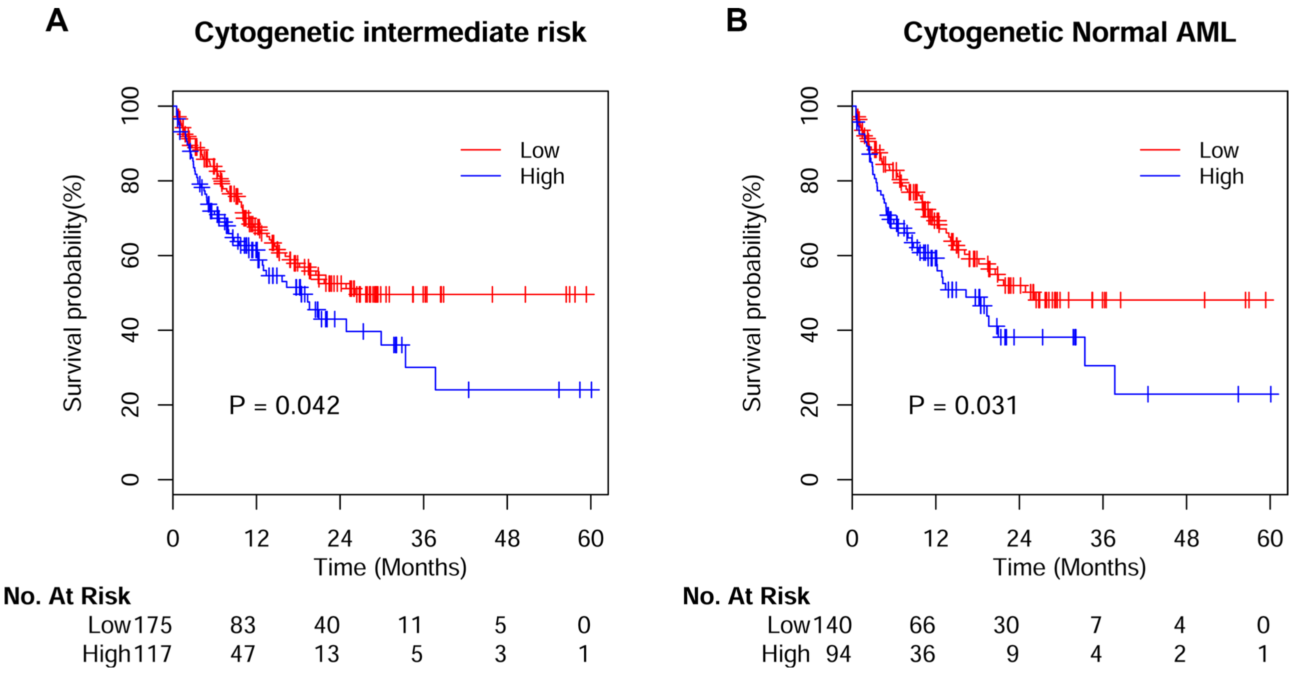

Supplementary Figure S1: Kaplan-Meier survival analysis of AML patients. Overall survival curves of the cytogenetically intermediate risk (**A**) and cytogenetically normal (**B**) AML patients in ZIH cohort according to distinct expression value of *MYH9* gene.

Supplementary Table S1: Multivariable analysis for overall survival in AML patients from TCGA cohort

Variables	HR (95% CI)	*P* value
MYH9 expression (high vs.low)	1.69 (1.17,2.43)	0.005
Age	1.04 (1.03,1.06)	< 0.001
Sex	0.74 (0.52,1.06)	0.097
WBC[1]	1.003 (0.999,1.007)	0.143
Percent blast	1.00 (0.99,1.01)	0.654
Karyotype		
Intermediate vs. favorable	2.49 (1.29,4.82)	0.007
Poor vs. favorable	4.31 (2.19,8.52)	< 0.001
CytoND vs .favorable	7.83 (2.42,25.37)	0.001
Gene mutations		
*FLT3*ITD	1.7 (1.06,2.72)	0.029
NPM1	0.72 (0.43,1.21)	0.215
CEBPA[dm2]	1.28 (0.76,2.15)	0.348

Abbreviations: [1]WBC,white blood cell; [2]DM: Double-allele. CI, confidence intervals; HR, hazard ratio, cytoND, karyotype is not determined.
